# 4-Chloro-*N*′-[(*E*)-2-chloro­benzyl­idene]benzohydrazide monohydrate

**DOI:** 10.1107/S1600536814008885

**Published:** 2014-04-26

**Authors:** Joel T. Mague, Shaaban K. Mohamed, Mehmet Akkurt, Herman Potgieter, Mustafa R. Albayati

**Affiliations:** a Department of Chemistry, Tulane University, New Orleans, LA 70118, USA; b Chemistry and Environmental Division, Manchester Metropolitan University, Manchester M1 5GD, England; c Chemistry Department, Faculty of Science, Minia University, 61519 El-Minia, Egypt; d Department of Physics, Faculty of Sciences, Erciyes University, 38039 Kayseri, Turkey; e Analytical Development Division, Manchester Metropolitan University, Manchester M1 5GD, England; f Kirkuk University, College of Science, Department of Chemistry, Kirkuk, Iraq

## Abstract

The title compound, C_14_H_10_Cl_2_N_2_O·H_2_O, has a nearly planar extended conformation [C—N—N—C = −173.66 (15)°]. The dihedral angle between the aromatic rings is 4.6 (2)°. The water mol­ecules alternate with benzohydrazide mol­ecules in chains formed by O—H⋯O hydrogen bonds which run parallel to the *a* axis. These chains are linked to neighboring chains through N—H⋯O and C—H⋯O inter­actions, forming a layer parallel to (001).

## Related literature   

For the biological activity of hydrazone compounds, see: Koopaei *et al.* (2013 ▶); Almasirad *et al.* (2005 ▶, 2006 ▶). For a similar structure, see: Cao (2009 ▶).
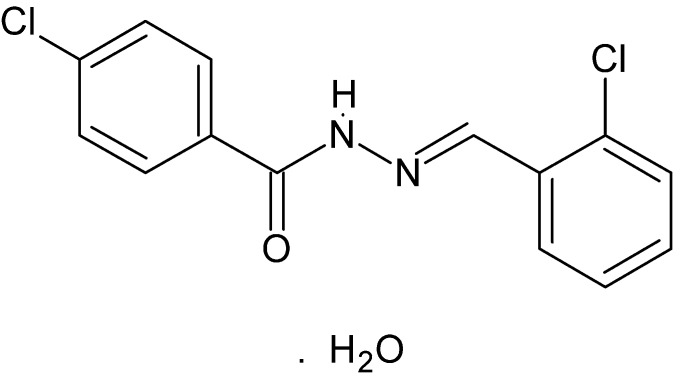


## Experimental   

### 

#### Crystal data  

C_14_H_10_Cl_2_N_2_O·H_2_O
*M* *_r_* = 311.16
Monoclinic, 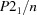

*a* = 4.6160 (5) Å
*b* = 12.8664 (15) Å
*c* = 23.681 (3) Å
β = 92.6760 (17)°
*V* = 1404.9 (3) Å^3^
*Z* = 4
Mo *K*α radiation
μ = 0.46 mm^−1^
*T* = 150 K
0.17 × 0.08 × 0.06 mm

#### Data collection  

Bruker SMART APEX CCD diffractometer
Absorption correction: multi-scan (*SADABS*; Bruker, 2013 ▶) *T*_min_ = 0.78, *T*_max_ = 0.97
24861 measured reflections
3511 independent reflections
2604 reflections with *I* > 2σ(*I*)
*R*_int_ = 0.064

#### Refinement  

*R*[*F* ^2^ > 2σ(*F* ^2^)] = 0.039
*wR*(*F* ^2^) = 0.097
*S* = 1.02
3511 reflections
181 parameters
H-atom parameters constrained
Δρ_max_ = 0.31 e Å^−3^
Δρ_min_ = −0.29 e Å^−3^

### 

Data collection: *APEX2* (Bruker, 2013 ▶); cell refinement: *SAINT* (Bruker, 2013 ▶); data reduction: *SAINT*; program(s) used to solve structure: *SHELXS97* (Sheldrick, 2008 ▶); program(s) used to refine structure: *SHELXL2014* (Sheldrick, 2008 ▶); molecular graphics: *DIAMOND* (Brandenburg & Putz, 2012 ▶); software used to prepare material for publication: *SHELXTL* (Sheldrick, 2008 ▶).

## Supplementary Material

Crystal structure: contains datablock(s) global, I. DOI: 10.1107/S1600536814008885/rz5119sup1.cif

Structure factors: contains datablock(s) I. DOI: 10.1107/S1600536814008885/rz5119Isup2.hkl

Supporting information file. DOI: 10.1107/S1600536814008885/rz5119Isup3.cml

CCDC reference: 998358

Additional supporting information:  crystallographic information; 3D view; checkCIF report

## Figures and Tables

**Table 1:** Hydrogen-bond geometry (Å, °)

*D*—H⋯*A*	*D*—H	H⋯*A*	*D*⋯*A*	*D*—H⋯*A*
N1—H1⋯O2^i^	0.91	1.95	2.8510 (19)	168
O2—H2*A*⋯O1^ii^	0.84	1.93	2.7632 (19)	172
O2—H2*B*⋯O1	0.84	1.95	2.7864 (19)	172
C2—H2⋯O2^i^	0.95	2.43	3.286 (2)	150
C8—H8⋯O2^i^	0.95	2.44	3.242 (2)	143

Symmetry codes: (i) 
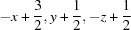
; (ii) 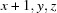.

**Fig. 1.**
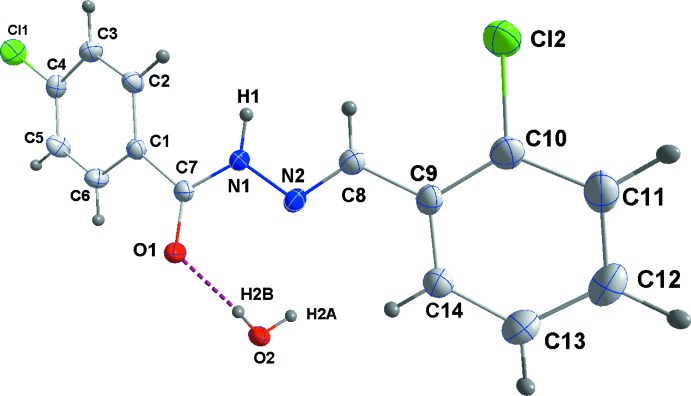
The asymmetric unit of the title compound showing one of the O—H···O interactions as a dotted line. Displacement ellipsoids are drawn at the 50% probability level.

**Fig. 2.**
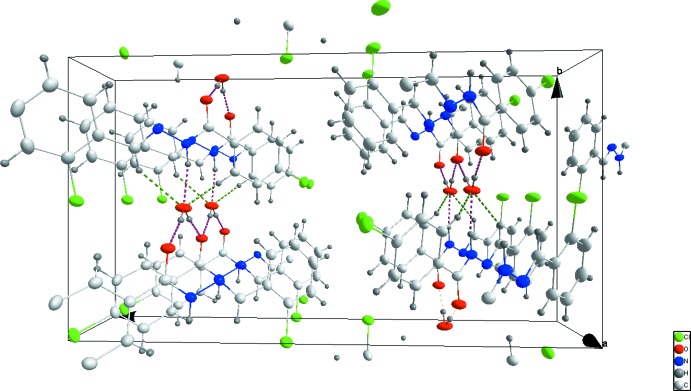
Packing of the title compound viewed down the a axis showing hydrogen interactions as dotted lines.

## supplementary crystallographic information

### 1. Comment

Use of non-steroidal anti-inflammatory drugs (NSAIDs) in treatment of pain and inflammation is usually associated with undesirable side effect such as gastrointestinal toxins and ulceration. Recently arylhydrazone scaffold compounds have showed safer profiles of activity and enhanced efficacy in the battle of pain in inflammatory diseases (Koopaei *et al.*, 2013). They have been depicted as dual COX/5-LO inhibitors (Almasirad *et al.*, 2005; Almasirad *et al.*, 2006). In this context and as part of our on-going study in the synthesis of safe profiles of anti-inflammatory pro-drugs we report the synthesis and crystal structure of the title compound.

The title compound (I) in Fig. 1 is in the "*extended*" conformation with the ring C1–C6 making a dihedral angle of 15.3 (1)° with the mean plane of the C1/C7/N1/O1 unit while the ring C9–C14 makes a dihedral angle of 4.6 (2)° with the plane of the C8/C9/N2 unit. The bond lengths and angles of (I) are normal and comparable with those observed for a similar compound (Cao, 2009).

The lattice water molecules alternate with molecules of (I) in chains formed by O2—H2A(or B)···O1 hydrogen bonds (Table 1 and Fig. 2) which run parallel to the *a* axis. These chains are linked to neighboring chains through N1—H1···O2 and C8—H8···O2 interactions. In these, the mean plane of the benzohydrazide molecule is inclined approximately 48° to (110).

### 2. Experimental

The compound was prepared by refluxing a mixture of 4-chlorobenzohydrazide (1 mmol, 171 mg)) with 2-chlorobenzaldehyde (1 mmol, 141 mg) in ethanol (30 mL) for 5h in the presence of a catalytic amount of glacial acetic acid. The mixture was cooled and the precipitate was filtered off, dried and recrystallized from ethanol to give pale brown crystals of poor quality. Slow evaporation of an aqueous ethanolic solution of the product afforded colorless block-like crystals of sufficient quality for x-ray diffraction. M. p. 452 454 K

### 3. Refinement

H-atoms attached to carbon were placed in calculated positions (C—H = 0.95 Å) while those attached to nitrogen and oxygen were placed in locations derived from a difference Fourier map and initially refined independently to ensure their initial positions were valid. In the final refinement, their coordinates adjusted to give N—H = 0.91 and O—H = 0.84 Å. All hydrogen atoms were then included as riding contributions with isotropic displacement parameters 1.2 times those of the attached atoms.

### Crystal data

**Table:**

C_14_H_10_Cl_2_N_2_O·H_2_O	*F*(000) = 640
*M**_r_* = 311.16	*D*_x_ = 1.471 Mg m^−^^3^
Monoclinic, *P*2_1_/*n*	Mo *K*α radiation, λ = 0.71073 Å
Hall symbol: -P 2yn	Cell parameters from 7842 reflections
*a* = 4.6160 (5) Å	θ = 2.3–28.1°
*b* = 12.8664 (15) Å	µ = 0.46 mm^−^^1^
*c* = 23.681 (3) Å	*T* = 150 K
β = 92.6760 (17)°	Column, colourless
*V* = 1404.9 (3) Å^3^	0.17 × 0.08 × 0.06 mm
*Z* = 4	

### Data collection

**Table:**

Bruker SMART APEX CCD diffractometer	3511 independent reflections
Radiation source: fine-focus sealed tube	2604 reflections with *I* > 2σ(*I*)
Graphite monochromator	*R*_int_ = 0.064
Detector resolution: 8.3660 pixels mm^-1^	θ_max_ = 28.4°, θ_min_ = 1.8°
φ and ω scans	*h* = −6→6
Absorption correction: multi-scan (*SADABS*; Bruker, 2013)	*k* = −16→17
*T*_min_ = 0.78, *T*_max_ = 0.97	*l* = −31→31
24861 measured reflections	

### Refinement

**Table:**

Refinement on *F*^2^	Primary atom site location: structure-invariant direct methods
Least-squares matrix: full	Secondary atom site location: difference Fourier map
*R*[*F*^2^ > 2σ(*F*^2^)] = 0.039	Hydrogen site location: mixed
*wR*(*F*^2^) = 0.097	H-atom parameters constrained
*S* = 1.02	*w* = 1/[σ^2^(*F*_o_^2^) + (0.0341*P*)^2^ + 0.6801*P*] where *P* = (*F*_o_^2^ + 2*F*_c_^2^)/3
3511 reflections	(Δ/σ)_max_ = 0.001
181 parameters	Δρ_max_ = 0.31 e Å^−^^3^
0 restraints	Δρ_min_ = −0.29 e Å^−^^3^

### Special details

**Table:**

Geometry. Bond distances, angles *etc*. have been calculated using the rounded fractional coordinates. All su's are estimated from the variances of the (full) variance-covariance matrix. The cell e.s.d.'s are taken into account in the estimation of distances, angles and torsion angles
Refinement. Refinement on *F*^2^ for ALL reflections except those flagged by the user for potential systematic errors. Weighted *R*-factors *wR* and all goodnesses of fit *S* are based on *F*^2^, conventional *R*-factors *R* are based on *F*, with *F* set to zero for negative *F*^2^. The observed criterion of *F*^2^ > σ(*F*^2^) is used only for calculating -*R*-factor-obs *etc*. and is not relevant to the choice of reflections for refinement. *R*-factors based on *F*^2^ are statistically about twice as large as those based on *F*, and *R*-factors based on ALL data will be even larger.

### Fractional atomic coordinates and isotropic or equivalent isotropic displacement parameters (Å^2^)

**Table:**

	*x*	*y*	*z*	*U*_iso_*/*U*_eq_	
Cl1	−0.46090 (11)	0.93219 (4)	0.06216 (2)	0.0343 (2)	
Cl2	1.10272 (13)	1.00504 (4)	0.41832 (2)	0.0434 (2)	
O1	0.4046 (3)	0.64950 (9)	0.23672 (6)	0.0271 (4)	
N1	0.5525 (3)	0.80924 (11)	0.26635 (6)	0.0213 (4)	
N2	0.7522 (3)	0.76724 (11)	0.30567 (6)	0.0223 (4)	
C1	0.1777 (3)	0.79601 (13)	0.19187 (7)	0.0195 (5)	
C2	0.0963 (4)	0.90021 (13)	0.19483 (8)	0.0229 (5)	
C3	−0.1015 (4)	0.94238 (14)	0.15527 (8)	0.0246 (5)	
C4	−0.2175 (4)	0.87909 (14)	0.11261 (7)	0.0228 (5)	
C5	−0.1440 (4)	0.77569 (14)	0.10916 (8)	0.0267 (6)	
C6	0.0548 (4)	0.73438 (14)	0.14875 (8)	0.0248 (5)	
C7	0.3874 (3)	0.74541 (13)	0.23339 (7)	0.0202 (5)	
C8	0.8769 (4)	0.83478 (14)	0.33826 (7)	0.0235 (5)	
C9	1.0970 (4)	0.80330 (14)	0.38149 (7)	0.0231 (5)	
C10	1.2181 (4)	0.87532 (15)	0.41988 (8)	0.0277 (6)	
C11	1.4287 (4)	0.84754 (16)	0.46082 (8)	0.0337 (6)	
C12	1.5197 (4)	0.74555 (17)	0.46427 (8)	0.0338 (6)	
C13	1.4015 (4)	0.67216 (16)	0.42740 (8)	0.0315 (6)	
C14	1.1943 (4)	0.70056 (15)	0.38619 (8)	0.0266 (6)	
O2	0.9062 (3)	0.53028 (10)	0.23079 (6)	0.0339 (4)	
H1	0.54250	0.87980	0.26400	0.0260*	
H2	0.17730	0.94280	0.22430	0.0280*	
H3	−0.15640	1.01340	0.15740	0.0300*	
H5	−0.22840	0.73310	0.08000	0.0320*	
H6	0.10770	0.66320	0.14640	0.0300*	
H8	0.82620	0.90610	0.33440	0.0280*	
H11	1.50920	0.89820	0.48620	0.0400*	
H12	1.66410	0.72570	0.49210	0.0410*	
H13	1.46280	0.60180	0.43030	0.0380*	
H14	1.11690	0.64950	0.36070	0.0320*	
H2A	1.04760	0.57140	0.23300	0.0410*	
H2B	0.76260	0.56910	0.23510	0.0410*	

### Atomic displacement parameters (Å^2^)

**Table:**

	*U*^11^	*U*^22^	*U*^33^	*U*^12^	*U*^13^	*U*^23^
Cl1	0.0347 (3)	0.0399 (3)	0.0273 (3)	0.0029 (2)	−0.0098 (2)	0.0061 (2)
Cl2	0.0618 (4)	0.0240 (3)	0.0424 (3)	−0.0030 (2)	−0.0175 (3)	−0.0013 (2)
O1	0.0226 (6)	0.0179 (6)	0.0401 (8)	0.0003 (5)	−0.0062 (5)	0.0002 (5)
N1	0.0193 (7)	0.0184 (7)	0.0258 (8)	0.0001 (5)	−0.0042 (6)	0.0022 (6)
N2	0.0193 (7)	0.0230 (8)	0.0242 (8)	0.0020 (6)	−0.0027 (6)	0.0039 (6)
C1	0.0179 (8)	0.0201 (8)	0.0206 (9)	−0.0016 (6)	0.0013 (6)	0.0017 (7)
C2	0.0244 (9)	0.0201 (8)	0.0240 (9)	−0.0011 (7)	−0.0024 (7)	−0.0016 (7)
C3	0.0249 (9)	0.0217 (9)	0.0272 (10)	0.0027 (7)	0.0000 (7)	0.0015 (7)
C4	0.0202 (8)	0.0295 (9)	0.0186 (9)	−0.0006 (7)	0.0001 (7)	0.0041 (7)
C5	0.0326 (10)	0.0268 (10)	0.0203 (9)	−0.0043 (8)	−0.0035 (7)	−0.0030 (7)
C6	0.0288 (9)	0.0208 (9)	0.0247 (9)	0.0001 (7)	0.0009 (7)	−0.0020 (7)
C7	0.0166 (8)	0.0192 (8)	0.0251 (9)	−0.0010 (6)	0.0028 (6)	0.0003 (7)
C8	0.0233 (9)	0.0228 (9)	0.0243 (9)	0.0000 (7)	−0.0004 (7)	0.0018 (7)
C9	0.0202 (8)	0.0265 (9)	0.0223 (9)	−0.0036 (7)	−0.0009 (7)	0.0027 (7)
C10	0.0308 (10)	0.0265 (10)	0.0256 (10)	−0.0037 (7)	−0.0018 (8)	0.0043 (8)
C11	0.0366 (11)	0.0383 (11)	0.0252 (10)	−0.0077 (9)	−0.0082 (8)	0.0010 (9)
C12	0.0294 (10)	0.0461 (12)	0.0252 (10)	0.0031 (9)	−0.0055 (8)	0.0089 (9)
C13	0.0305 (10)	0.0340 (11)	0.0298 (11)	0.0069 (8)	−0.0003 (8)	0.0058 (8)
C14	0.0263 (9)	0.0272 (10)	0.0261 (10)	−0.0004 (7)	−0.0011 (7)	0.0014 (8)
O2	0.0232 (6)	0.0178 (6)	0.0601 (10)	−0.0009 (5)	−0.0037 (6)	0.0041 (6)

### Geometric parameters (Å, º)

**Table:**

Cl1—C4	1.7410 (18)	C8—C9	1.465 (2)
Cl2—C10	1.752 (2)	C9—C10	1.397 (3)
O1—C7	1.239 (2)	C9—C14	1.399 (3)
O2—H2B	0.8400	C10—C11	1.387 (3)
O2—H2A	0.8400	C11—C12	1.379 (3)
N1—C7	1.345 (2)	C12—C13	1.381 (3)
N1—N2	1.389 (2)	C13—C14	1.383 (3)
N2—C8	1.281 (2)	C2—H2	0.9500
N1—H1	0.9100	C3—H3	0.9500
C1—C6	1.393 (2)	C5—H5	0.9500
C1—C2	1.395 (2)	C6—H6	0.9500
C1—C7	1.496 (2)	C8—H8	0.9500
C2—C3	1.388 (3)	C11—H11	0.9500
C3—C4	1.386 (3)	C12—H12	0.9500
C4—C5	1.376 (3)	C13—H13	0.9500
C5—C6	1.387 (3)	C14—H14	0.9500
			
H2A—O2—H2B	103.00	Cl2—C10—C11	117.56 (15)
N2—N1—C7	119.47 (14)	C10—C11—C12	119.22 (18)
N1—N2—C8	113.93 (14)	C11—C12—C13	120.19 (18)
N2—N1—H1	117.00	C12—C13—C14	120.34 (19)
C7—N1—H1	123.00	C9—C14—C13	120.97 (18)
C2—C1—C7	123.55 (15)	C3—C2—H2	120.00
C2—C1—C6	118.86 (15)	C1—C2—H2	120.00
C6—C1—C7	117.58 (15)	C2—C3—H3	121.00
C1—C2—C3	120.81 (16)	C4—C3—H3	121.00
C2—C3—C4	118.82 (16)	C6—C5—H5	120.00
Cl1—C4—C3	119.00 (14)	C4—C5—H5	120.00
C3—C4—C5	121.56 (17)	C1—C6—H6	120.00
Cl1—C4—C5	119.44 (14)	C5—C6—H6	120.00
C4—C5—C6	119.18 (17)	C9—C8—H8	120.00
C1—C6—C5	120.77 (16)	N2—C8—H8	120.00
N1—C7—C1	116.57 (14)	C10—C11—H11	120.00
O1—C7—C1	120.85 (15)	C12—C11—H11	120.00
O1—C7—N1	122.58 (15)	C13—C12—H12	120.00
N2—C8—C9	120.72 (16)	C11—C12—H12	120.00
C10—C9—C14	117.26 (16)	C12—C13—H13	120.00
C8—C9—C14	121.76 (16)	C14—C13—H13	120.00
C8—C9—C10	120.99 (16)	C9—C14—H14	120.00
Cl2—C10—C9	120.42 (14)	C13—C14—H14	120.00
C9—C10—C11	122.01 (18)		
			
C7—N1—N2—C8	−173.66 (15)	C3—C4—C5—C6	−1.2 (3)
N2—N1—C7—O1	0.3 (2)	C4—C5—C6—C1	0.5 (3)
N2—N1—C7—C1	−179.81 (13)	N2—C8—C9—C10	−175.19 (17)
N1—N2—C8—C9	−178.99 (15)	N2—C8—C9—C14	4.7 (3)
C6—C1—C2—C3	−0.6 (3)	C8—C9—C10—Cl2	1.6 (2)
C7—C1—C2—C3	−179.41 (16)	C8—C9—C10—C11	−179.38 (17)
C2—C1—C6—C5	0.4 (3)	C14—C9—C10—Cl2	−178.37 (14)
C7—C1—C6—C5	179.20 (16)	C14—C9—C10—C11	0.7 (3)
C2—C1—C7—O1	163.87 (16)	C8—C9—C14—C13	−179.76 (17)
C2—C1—C7—N1	−16.0 (2)	C10—C9—C14—C13	0.2 (3)
C6—C1—C7—O1	−14.9 (2)	Cl2—C10—C11—C12	178.38 (15)
C6—C1—C7—N1	165.23 (15)	C9—C10—C11—C12	−0.7 (3)
C1—C2—C3—C4	0.0 (3)	C10—C11—C12—C13	−0.1 (3)
C2—C3—C4—Cl1	−178.89 (14)	C11—C12—C13—C14	1.0 (3)
C2—C3—C4—C5	0.9 (3)	C12—C13—C14—C9	−1.0 (3)
Cl1—C4—C5—C6	178.61 (14)		

### Hydrogen-bond geometry (Å, º)

**Table:**

*D*—H···*A*	*D*—H	H···*A*	*D*···*A*	*D*—H···*A*
N1—H1···O2^i^	0.91	1.95	2.8510 (19)	168
O2—H2*A*···O1^ii^	0.84	1.93	2.7632 (19)	172
O2—H2*B*···O1	0.84	1.95	2.7864 (19)	172
C2—H2···O2^i^	0.95	2.43	3.286 (2)	150
C8—H8···O2^i^	0.95	2.44	3.242 (2)	143

Symmetry codes: (i) −*x*+3/2, *y*+1/2, −*z*+1/2; (ii) *x*+1, *y*, *z*.
